# Delta oscillations phase limit neural activity during sevoflurane anesthesia

**DOI:** 10.1038/s42003-019-0664-3

**Published:** 2019-11-15

**Authors:** Shubham Chamadia, Juan C. Pedemonte, Eunice Y. Hahm, Jennifer Mekonnen, Reine Ibala, Jacob Gitlin, Breanna R. Ethridge, Jason Qu, Rafael Vazquez, James Rhee, Erika T. Liao, Emery N. Brown, Oluwaseun Akeju

**Affiliations:** 1 Department of Anesthesia, Critical Care and Pain Medicine, Massachusetts General Hospital, Harvard Medical School, Boston, MA 02114 USA; 2 División de Anestesiología, Escuela de Medicina, Pontificia Universidad Católica de Chile, Santiago, Chile; 3 Tulane University of Medicine, New Orleans, LA 70112 USA; 4 Department of Brain and Cognitive Science, Institute for Medical Engineering and Sciences, Picower Institute for Learning and Memory, Institute for Data Systems and Society, Massachusetts Institute of Technology, Cambridge, MA 02139 USA; 5 McCance Center for Brain Health, Massachusetts General Hospital, Harvard Medical School, Boston, MA 02114 USA

**Keywords:** Neural circuits, Neurophysiology

## Abstract

Understanding anesthetic mechanisms with the goal of producing anesthetic states with limited systemic side effects is a major objective of neuroscience research in anesthesiology. Coherent frontal alpha oscillations have been postulated as a mechanism of sevoflurane general anesthesia. This postulate remains unproven. Therefore, we performed a single-site, randomized, cross-over, high-density electroencephalogram study of sevoflurane and sevoflurane-*plus*-ketamine general anesthesia in 12 healthy subjects. Data were analyzed with multitaper spectral, global coherence, cross-frequency coupling, and phase-dependent methods. Our results suggest that coherent alpha oscillations are not fundamental for maintaining sevoflurane general anesthesia. Taken together, our results suggest that subanesthetic and general anesthetic sevoflurane brain states emerge from impaired information processing instantiated by a delta-higher frequency phase-amplitude coupling syntax. These results provide fundamental new insights into the neural circuit mechanisms of sevoflurane anesthesia and suggest that anesthetic states may be produced by extracranial perturbations that cause delta-higher frequency phase-amplitude interactions.

## Abstract (web summary)

Chamadia et al. show that a delta-higher frequency phase-amplitude coupling syntax, rather than coherent frontal alpha oscillations, is fundamental to sevoflurane anesthesia. Their conclusion is based on analyzing different EEG measures across different anesthetic states.

## Introduction

General anesthesia is a drug-induced reversible state of unconsciousness, amnesia, antinociception, and immobility, with the maintenance of physiological stability^1^. Since the first public demonstration of diethyl ether as a general anesthetic occurred at the Massachusetts General Hospital (MGH) in 1846, the practice of anesthesiology has revolutionized surgery and spurred advances in modern medicine. Now, over 170 years later, the administration of diethyl ether derivatives remains fundamental to anesthesiology. Fluoromethyl hexafluoroisopropyl ether, also known as sevoflurane, is a derivative of diethyl ether that is extensively used in current clinical practice. Basic science studies have helped to identify the molecular targets of sevoflurane. These targets include γ-aminobutyric acid type A (GABA_A_) receptors, glycine receptors, two-pore potassium channels, and *N*-methyl-d-aspartate receptors, among many others^2–5^. However, a significant knowledge gap exists in relating how drug activity at molecular targets results in the neural circuit perturbations that cause anesthetic states.

Neural oscillations are a prominent feature of the anesthetized brain^1^. These oscillations change systematically as a function of anesthetic drug dose^6–11^ and age^12–15^, which suggests that they are generated from mechanisms that depend on cellular properties such as ionic currents, myelin integrity, and synaptic density. Neural oscillations are important for information processing in the brain^16–19^. Thus, anesthetic-drug-induced oscillations may disrupt the brain’s capacity for information processing in cognitive, memory and sensory circuits. Coherent frontal alpha oscillations—an oscillatory dynamic associated with sevoflurane^10^ and the other modern-day derivatives of ether anesthesia^20^—have been postulated as a fundamental mechanism by which the derivatives of diethyl ether cause unconsciousness^10^. This postulate remains unproven, and many sevoflurane general anesthesia scenarios without coherent alpha oscillations have been identified. For example, alpha oscillations are not evident in the electroencephalogram (EEG) of patients until approximately 4 months of age and do not become coherent until approximately 10 months of age^15^. Thus, the neural circuit mechanisms of sevoflurane general anesthesia are not clear.

To formally evaluate neural circuit mechanisms underlying sevoflurane general anesthesia, we conducted a study of sevoflurane anesthetic states in healthy human volunteers. Human neurophysiological studies of anesthetic-drug-induced states have typically used the loss of behavioral responsiveness to nonsalient stimuli, such as auditory cues, as a shorthand for unconsciousness. This experimentally convenient approach may engender considerable confusion because the loss of responsiveness occurs during subanesthetic states that do not approximate general anesthesia. Further, states of internal processing such as dreaming may persist during subanesthetic states^21,22^. We aimed to obtain fundamental new insights into anesthetic mechanisms of sevoflurane by targeting and studying subanesthetic and general anesthetic brain states using sevoflurane anesthetic concentrations that are consistent with current clinical practice and epidemiologically based characterizations^23,24^.

Sevoflurane-induced lack of responsiveness is associated with loss of occipitally dominant alpha oscillations prior to the occurrence of frontal alpha  oscillations^25^. Here, we investigated neural circuit dynamics to explain sevoflurane-induced subanesthetic, general anesthetic, and deep general anesthetic brain states using high-density EEG recordings. We have previously suggested that ketamine, an anesthetic drug that is typically administered as an adjunct for antinociception during general anesthesia, is associated with decreased alpha oscillation power and coherence^20^. Therefore, in a separate experiment, we specifically tested the hypothesis that coherent alpha oscillations are fundamental for sevoflurane general anesthesia by studying the effects of ketamine on sevoflurane-induced oscillations. Previously described neural correlates of propofol general anesthesia are based on the amplitude (power), frequency-dependent correlation, and phase−amplitude dynamics of neural oscillations^6,11^. Therefore, we implemented multitaper spectral analysis to characterize the amplitude (power), global coherence analysis to characterize frequency-dependent correlation structure, and nonlinear cross-frequency coupling analyses to characterize the phase−amplitude dynamics of sevoflurane-induced oscillations. We also aimed to study whether dexmedetomidine, an anesthetic drug that modulates the alpha-2a receptor, shares neural correlates that approximate those of sevoflurane.

## Results

**Fig. 1:**
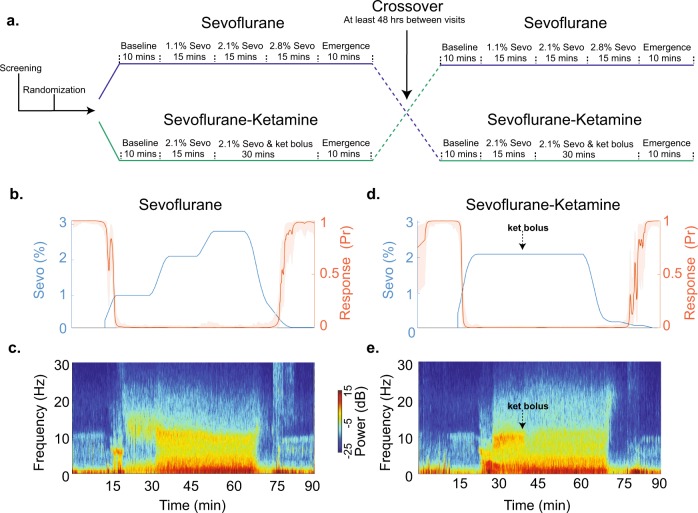
Schematic of the study protocol and data from an illustrative subject. **a** We acquired data using a randomized, cross-over study design. Anesthetic-drugs were administered for approximately 45 min. **b**, **c** End-tidal sevoflurane concentration, behavioral response probability curve, and corresponding frontal electroencephalogram spectrogram during the sevoflurane study visit. **d**, **e** End-tidal sevoflurane concentration, behavioral response probability curve, and corresponding frontal electroencephalogram spectrogram during the sevoflurane-plus-ketamine study visit. Shaded regions represent the 95% confidence bounds. Sevo sevoflurane, Ket Ketamine

No adverse events were reported during this study. Figure 1a is the schematic of our randomized, cross-over study design. Figure 1b–e illustrates data obtained from a subject during sevoflurane and sevoflurane-plus-ketamine study visits. In this manuscript, we use the term anesthetic depth to refer to the increased level of brain hyperpolarization associated with increased anesthetic drug administration.

### Loss of responsiveness to behavioral stimuli is not synonymous with general anesthesia

We aligned the data (±10 min) to the probabilistic definition of LOR (loss of responsiveness) and ROR (return of responsiveness) (Supplementary Fig. 1). The end-tidal sevoflurane concentration at which LOR occurred was 1% (SD, 0.1). ROR occurred at 0.2% (0.1) (Supplementary Fig. 1a–d). During the sevoflurane-plus-ketamine visit in which sevoflurane was rapidly administered, the end-tidal sevoflurane concentration at which LOR occurred was 1.8% (0.2). ROR occurred at 0.2% (0.1) (Supplementary Fig. 1e–h). EEG oscillations during LOR were inconsistent with dynamics that have previously been described for sevoflurane general anesthesia^10^.

### Alpha oscillation power did not covary with anesthetic depth

**Fig. 2:**
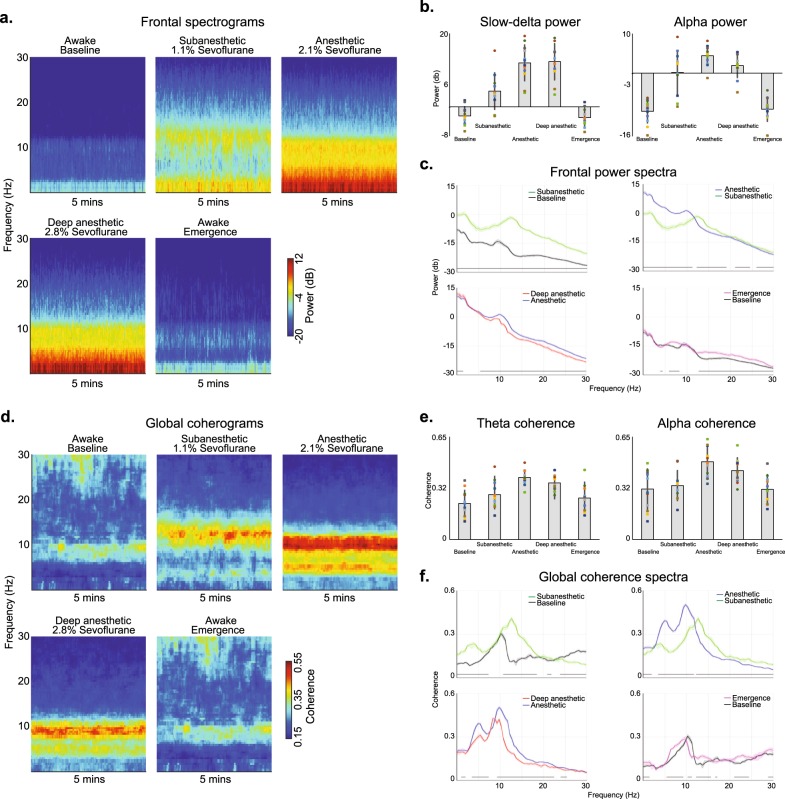
Spectral and global coherence analysis of the sevoflurane study visit. **a** The median frontal spectrograms demonstrate that electroencephalogram oscillations change systematically as a function of the anesthetic state. **b** Power in the canonical slow-delta and alpha frequency bands change with respect to the anesthetic states. **c** Frontal spectra and bootstrapped difference of median spectra confirm that electroencephalogram oscillations change systematically as a function of the anesthetic state. Increases in slow oscillation power covaried with anesthetic depth. Alpha oscillation power did not covary with the anesthetic state. **d** The median global coherograms demonstrate that global coherence also changes systematically as a function of the anesthetic state. Globally coherent beta oscillations were associated with the subanesthetic state, while globally coherent alpha and theta oscillations were associated with the anesthetic states. Globally coherent theta and alpha oscillations did not covary with the anesthetic state. **e** Global coherence in the canonical theta and alpha frequency bands change with respect to the anesthetic states. **f** Global coherence spectra and bootstrapped difference of median global coherence confirm that theta and alpha global coherence did not covary with the anesthetic state. Shaded regions represent the 99% confidence bounds of the bootstrapped median power spectra. Black lines represent frequency bands that met our threshold for statistical significance

We analyzed the neural oscillations associated with awake (baseline), subanesthetic (1.1% sevoflurane), anesthetic (2.1% sevoflurane), deep anesthetic (2.8% sevoflurane), and awake (emergence) states. We computed frontal spectrograms (Fig. 2a), power within canonical frequency bands of interest (Fig. 2b), and spectra differences (Fig. 2c). We also computed occipital spectrograms (Supplementary Fig. 2a), spectra differences (Supplementary Fig. 2b), and power spectral-spatial plots (Supplementary Fig. 2c).

When we analyzed data from frontal electrodes, we found that alpha oscillations did not covary with increasing anesthetic depth, such that EEG power was increased during the anesthetic state but decreased during the deep anesthetic state. However, slow oscillation power covaried with anesthetic depth. We implemented a noncanonical frequency band-based bootstrap procedure for statistical inference. These data are summarized in Fig. 2c. Data from occipital electrodes are summarized in Supplementary Fig. 2b.

### Globally coherent alpha oscillation did not covary with anesthetic depth

Increased global coherence may occur despite decreases in oscillatory power. Therefore, we next analyzed the global coherence structure of the baseline, subanesthetic, anesthetic, deep anesthetic, and awake states. We computed global coherograms (Fig. 2d), global coherence within canonical frequency bands of interest (Fig. 2e), and global coherence differences (Fig. 2f). We also computed global coherence spatial plots (Supplementary Fig. 2d). We found that the anesthetic states were associated with globally coherent theta and alpha oscillations. However, these globally coherent oscillations did not covary with anesthetic depth such that coherence was increased during the anesthetic state but decreased during the deep anesthetic state. Supplementary Movie 1 illustrates the oscillatory and global coherence changes associated with sevoflurane anesthetic states.

### Ketamine induced relatively active electroencephalogram oscillations during sevoflurane general anesthesia

**Fig. 3:**
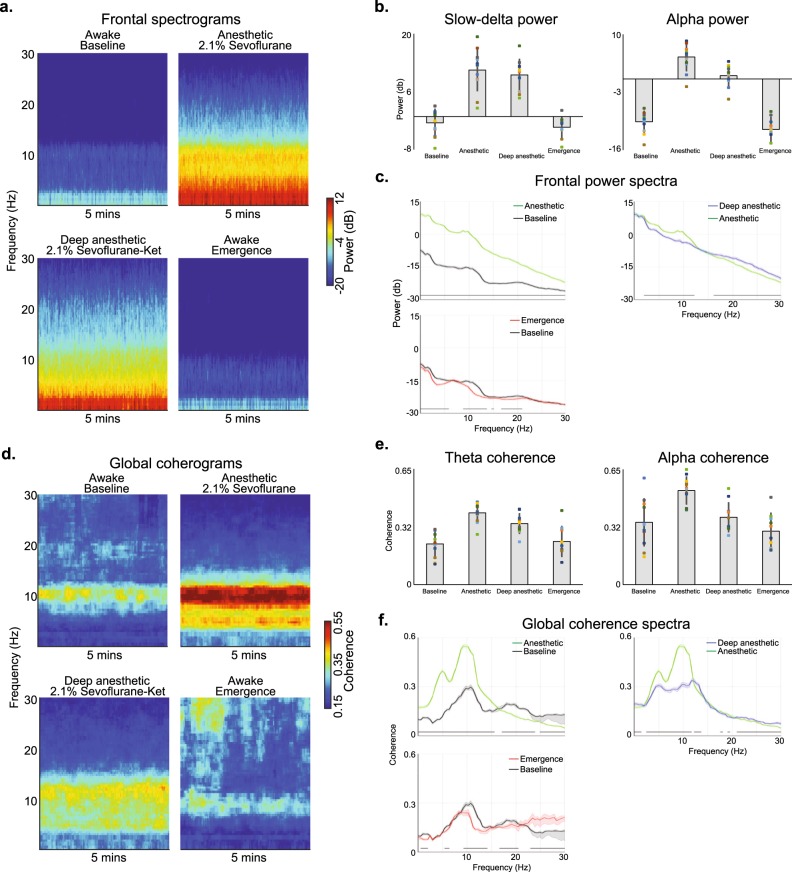
Spectral and global coherence analysis of the sevoflurane-plus-ketamine study visit. **a** Ketamine reduced the power of alpha oscillations during sevoflurane general anesthesia. **b** Power in the canonical slow-delta and alpha frequency bands change with respect to the anesthetic states. **c** Frontal spectra and bootstrapped difference of median spectra confirm that the ketamine-induced alpha oscillation power decrease was significant. Ketamine was also associated with a decrease in delta oscillation power and an increase in beta oscillation power. **d** The median global coherograms demonstrate ketamine reduced the global coherence of theta and alpha oscillations. **e** Global coherence in the canonical theta and alpha frequency bands change with respect to the anesthetic states. **f** Global coherence spectra and bootstrapped difference of median global coherence confirm that ketamine significantly reduced theta and alpha global coherence to suggest this dynamic is not fundamental for general anesthesia. Shaded regions represent the 99% confidence bounds of the bootstrapped median global coherence spectra. Black lines represent frequency bands that met our threshold for statistical significance

Next, we tested the hypothesis that coherent alpha oscillations are fundamental for sevoflurane general anesthesia by studying the effects of ketamine on sevoflurane-induced oscillations. We analyzed the neural oscillations associated with awake (baseline), anesthetic (2.1% sevoflurane), deep anesthetic (2.1% sevoflurane plus ketamine; SevoKet), and awake (emergence) states. We computed frontal spectrograms (Fig. 3a), power within canonical frequency bands of interest (Fig. 3b), and spectra differences (Fig. 3c). We also computed occipital spectrograms (Supplementary Fig. 3a), spectra differences (Supplementary Fig. 3b), and power spectral-spatial plots (Supplementary Fig. 3c).

When we analyzed data from frontal electrodes, we found that delta oscillation power was decreased during the SevoKet deep anesthetic state. The SevoKet deep anesthetic state was associated with a paradoxical increase in beta oscillation power. These data are also summarized in Fig. 3c. Data from occipital electrodes are summarized in Supplementary Fig. 3b.

### Ketamine reduced globally coherent oscillations during sevoflurane general anesthesia

We next analyzed the effect of ketamine on the global coherence structure of the baseline, anesthetic, SevoKet deep anesthetic, and awake states. We computed global coherograms (Fig. 3d), global coherence within canonical frequency bands of interest (Fig. 3e), and spectra differences (Fig. 3f). We also computed global coherence spatial plots (Supplementary Fig. 3d). We found that the SevoKet deep anesthetic state was associated with decreased globally coherent theta and alpha oscillations. Thus, globally coherent oscillations did not covary with anesthetic depth. These data are summarized in Fig. 3f. Supplementary Movie 2 illustrates the oscillatory and global coherence changes associated with sevoflurane anesthetic states.

### Sevoflurane anesthetic states were associated with a phase−amplitude coupling syntax

The power and global coherence of sevoflurane-induced oscillations did not track with anesthetic depth. Therefore, we next investigated whether anesthetic states emerge from impaired information processing instantiated by cross-frequency coupling.

**Fig. 4:**
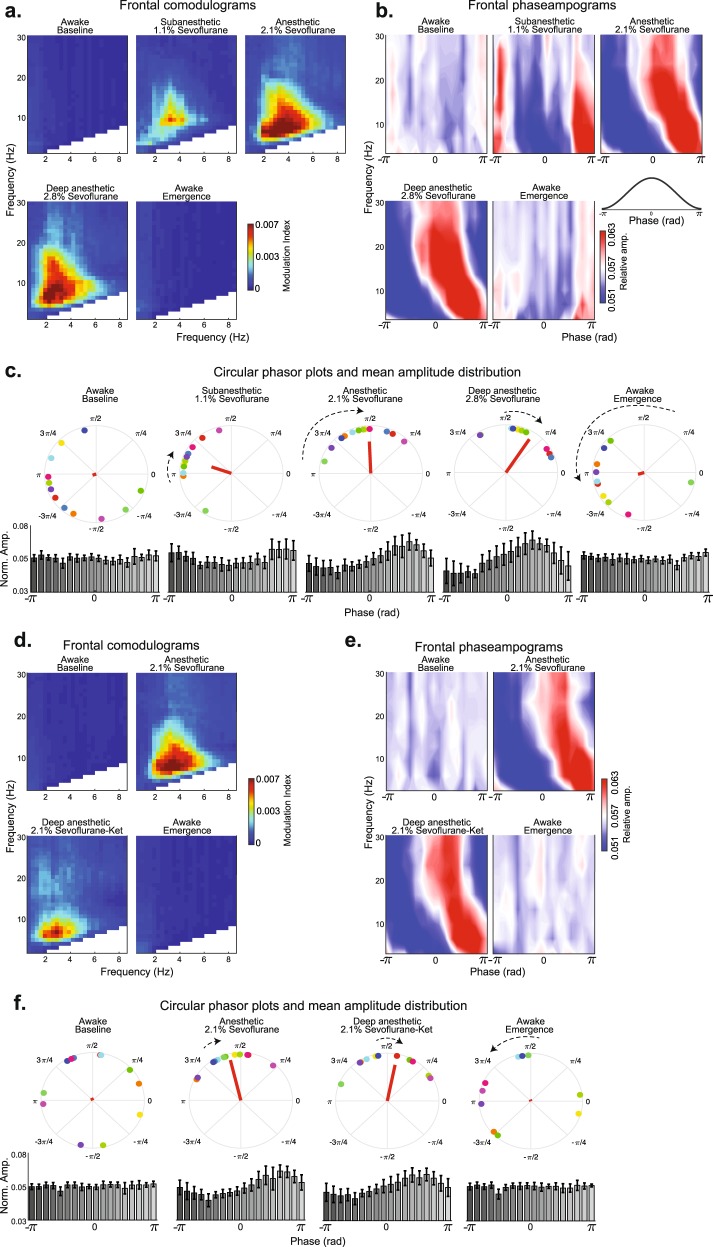
Phase-amplitude coupling dynamics associated with sevoflurane-induced anesthetic states. **a** Frontal comodulograms demonstrated that delta oscillations modulated higher frequencies during sevoflurane-induced anesthetic states. **b** Frontal phaseampograms between delta and higher frequencies demonstrated that distinct patterns of phase limited neural activity are associated with subanesthetic and anesthetic states. **c** Frontal circular phasor plots demonstrated that neural activity systematically shifted from π towards 0 phase of delta oscillations as a function of anesthetic depth. The median amplitude vector (red line) was increased from baseline during the anesthetic states. Mean amplitude distribution was not uniformly distributed during sevoflurane-induced anesthetic states. **d** Frontal comodulograms demonstrated that delta oscillations modulated higher frequencies during the sevoflurane-plus-ketamine induced anesthetic state. **e** Frontal phaseampograms between delta and higher frequencies demonstrated that the distinct patterns of phase limited neural activity associated with the sevoflurane general anesthetic state were conserved during the sevoflurane-plus-ketamine anesthetic state. **f** Frontal circular phasor plots also demonstrated that neural activity systematically shifted from π towards 0 phase of delta oscillations as a function of anesthetic depth. The median amplitude vector (red line) was increased from baseline during the anesthetic states. Mean amplitude distribution was not uniformly distributed during sevoflurane-induced anesthetic states. Colored circular dots on phasor plots represent subject level data. Error bars represent standard deviation

First, we computed comodulograms to elicit the putative phase driver(s) associated with sevoflurane anesthesia states. The comodulogram plots demonstrated that delta oscillation indices were highest during sevoflurane anesthetic states (Fig. 4a). The spatial representation of comodulograms is presented in Supplementary Fig. 4a. We next analyzed phase−amplitude coupling dynamics between delta and higher frequency oscillations by computing phaseampograms (Fig. 4b). We observed that the relative amplitude to higher-frequency oscillations was restricted to the ±π phase of delta oscillations during the subanesthetic state. The relative amplitude of oscillations <8 Hz remained restricted to the ±π phase of delta oscillations during sevoflurane general anesthesia. However, oscillations >8 Hz were restricted to approximately −π/3 to π/3 phase of delta oscillations. The relative amplitude of oscillations <6 Hz remained restricted to the ±π phase of delta oscillations during the deep general anesthetic state. However, oscillations >6 Hz were restricted to approximately −π/3 to π/3 phase of delta oscillations.

To enable statistical inference, we computed circular phasor plots (Fig. 4c; top panel) for phase driver (2–4 Hz) and higher frequency oscillations (13–20 Hz). We observed that the mean phase angle shifted from approximately π phase towards π/4 phase as a function of anesthetic depth (baseline at −9π/10; subanesthetic at −9π/10; anesthetic at π/2; deep anesthetic at π/3; emergence at −9π/10). The distribution of the circular mean of the mean amplitude vectors (Fig. 4c; bottom panel) was not uniformly distributed during the sevoflurane anesthesia states (baseline, *p* = 0.259; subanesthetic, *p* = 0.011; general anesthetic, *p* = 0.006; deep general anesthetic, *p* = 0.011; emergence, *p* = 0.156; omnibus test). We also observed that the mean amplitude vector for delta oscillation phase became larger as a function of anesthetic depth (subanesthetic > baseline, *p* = 0.104; general anesthetic > subanesthetic, *p* = 0.0171; linear mixed-effects model). The anesthetic and deep anesthetic comparison was not significantly different (*p* = 0.1367).

We also computed the spatial distribution of phaseampograms (Supplementary Fig. 5a) and circular phasor plots and mean amplitude histograms of slow and delta driver frequencies (Supplementary Fig. 5b, c). Worse signal to noise ratio can be observed in the mean amplitude distribution of the slow driver.

### Sevoflurane phase−amplitude coupling syntax tracks with the anesthetic state

We previously demonstrated that sevoflurane plus ketamine general anesthesia is associated with relatively activated EEG oscillations. Therefore, we computed comodulograms to assess whether the putative neural phase−amplitude syntax that we observed during sevoflurane general anesthesia was conserved after the administration of ketamine. The comodulogram plots demonstrated that delta oscillation modulation indices were highest during sevoflurane general anesthetic states (Fig. 4d). Spatial representation of comodulograms is presented in Supplementary Fig. 4b. We analyzed phase−amplitude coupling dynamics between delta and higher frequency oscillations by computing phaseampograms (Fig. 4e). Similar to the findings described above, the relative amplitude of oscillations <8 Hz was restricted to the ±π phase of delta oscillations during sevoflurane general anesthesia. However, oscillations >8 Hz were restricted to approximately −π/3 to π/3 phase of delta oscillations. Similar to findings described for the deep anesthetic state during sevoflurane visit, the relative amplitude of oscillations <6 Hz remained restricted to the ±π phase of delta oscillations while oscillations >8 Hz were restricted to approximately −π/3 to π/3 phase of delta oscillations during the SevoKet deep general anesthesia state.

We also computed circular phasor plots (Fig. 4f; top panel) for phase driver (2–4 Hz) and higher frequency oscillation (13–20 Hz). We observed that the mean phase angle shifted from approximately π phase towards π/4 phase as a function of anesthetic depth (baseline at 3π/5; anesthetic at 11π/20; deep anesthetic at 2π/5; emergence at 3π/5). The distribution of the circular mean of the mean amplitude vectors (Fig. 4f; bottom panel) was not uniformly distributed during the sevoflurane anesthesia states (baseline, *p* = 0.645; general anesthetic, *p* = 0.006; sevoKet deep general anesthetic, *p* = 0.012; emergence, *p* = 0.527; omnibus test). We also observed that the mean amplitude vector for delta oscillation phase became larger as a function of anesthetic depth (general anesthetic > baseline, *p* < 0.001; linear mixed-effects model). The anesthetic and SevoKet deep anesthetic state comparison was not significant (*p* = 0.9560).

We also computed the spatial distribution of phaseampograms (Supplementary Fig. 6a), and circular phasor plots and mean amplitude histograms of slow and delta driver frequencies (Supplementary Fig. 6b, c). Worse signal to noise ratio can be observed in the mean amplitude distribution of the slow driver.

### The phase−amplitude coupling syntax for subanesthetic states may be conserved across drug classes

Dexmedetomidine is an anesthetic drug that acts as an agonist at the alpha-2a receptor to induce a subanesthetic state. First, we computed comodulograms to elicit the putative phase driver(s) associated with the dexmedetomidine state. The comodulogram plots demonstrated that slow oscillation modulation indices were high during the dexmedetomidine state (Supplementary Fig. 7a). Spatial representation of comodulograms is presented in Supplementary Fig. 7b. We observed that the relative amplitude to higher frequency oscillations was restricted to the ±π phase of slow oscillations during the dexmedetomidine state in frontal channels. We also computed circular phasor plots (Supplementary Fig. 7c; top panel) for phase driver (0.1–1.5 Hz) and higher-frequency oscillation (13–16 Hz). The distribution of the circular mean of the mean amplitude vectors (Supplementary Fig. 7b; bottom panel) was not uniformly distributed during the dexmedetomidine anesthesia state (baseline, *p* = 0.376; subanesthetic, *p* = 0.0107; omnibus test).

## Discussion

Our findings demonstrate that sevoflurane sedation, a subanesthetic state from which patients can be aroused to consciousness, is associated with phase restricted activity of neural oscillations to the trough (π) region of delta oscillations. During sevoflurane general anesthesia states, the phase-restricted activity of ~0.1–6 Hz oscillations to the trough region was maintained. However, ~8–30 Hz frequency oscillations became restricted to the peak (~−π/3 to π/3) region of delta oscillations. This dynamic was maintained across all electrode sensor locations, although modulation indices were strongest in frontal electrode locations. The magnitude and phase of the mean normalized phase−amplitude coupling vector, not the power or global coherence of oscillations, tracked with anesthetic depth. Taken together, our results provide strong evidence that subanesthetic and general anesthetic brain states emerge from impaired information processing instantiated by a delta-higher frequency phase−amplitude coupling syntax.

At the neuronal level, slow oscillations are associated with an alternation between Up states where neurons are able to fire, and Down states where neurons are relatively silent^26,27^. General anesthesia is associated with longer Down states in humans^26^. The ionic currents underlying cortically generated delta oscillations are similar to those underlying the slow oscillation Up and Down states^28^. Neural activity during the Down state may result from intrinsic and synaptic properties of pyramidal cells in layer 5 such as the summation of spontaneous action potential independent excitatory synaptic potentials^29–32^ or from neurons that fire persistently during the Down state^33–36^. Neural activity during the Up state may result from enhanced activity of inhibitory interneurons and activation of activity-dependent hyperpolarizing currents^37–39^. Thus, the phase−amplitude coupling dynamics we describe suggest that membrane conductance and timescales governing well-timed neuronal activity are markedly prolonged by sevoflurane. Thus, the repertoire of possible neuronal firing and coupling dynamics are restricted to a rigid structure during anesthetic states. Neurophysiological recordings across various spatial scales are necessary to precisely define the micro-circuit dynamics underlying our findings.

A unifying mechanism to explain how anesthetic drugs from various drug classes produce general anesthesia may not be achievable. Theories of information processing propose that phase−amplitude coupling is critical for regulating neuronal activity and information processing across spatial and temporal scales^17,18,40,41^. Phase−amplitude coupling between slow oscillations and thalamocortical alpha oscillations was recently described for propofol anesthesia^6^. We also show that dexmedetomidine, an alpha-2a receptor agonist that patterns the activity of various arousal nuclei similar to sleep^1^, is associated with phase−amplitude coupling between slow and spindle oscillations. Thus, although the center frequency of phase drivers may change as a function of the anesthetic drug class or drug dose, we speculate that phase−amplitude coupling may be consistent across anesthetic states produced by various anesthetic drug classes. While our present work supports this conclusion, further research is required to more thoroughly explore this hypothesis.

Although functional imaging studies are not a surrogate for neuronal spiking activity, they provide a measure of the collective behavior of neurons. Functional imaging studies of subanesthetic sevoflurane and subanesthetic dexmedetomidine have previously demonstrated that cortico-cortical connectivity between brain regions that comprise the Default Mode Network is preserved during these states^21,42–45^. Thus, the phase−amplitude pattern we describe during the subanesthetic state may reflect functionally coupled cortical neural activity that is primed for the response to external stimuli. Conversely, sevoflurane general anesthesia is associated with impaired cortico-cortical connectivity between brain regions that comprise the Default Mode Network^43,46^. Thus, the phase−amplitude pattern we describe during this state may reflect functionally decoupled neural activity that is not receptive to external stimuli. We note that the phase−amplitude syntax we describe is consistent with asynchronous^26^ and hypersynchronous^47^ low-frequency oscillations causing altered arousal states. Further, our findings of decreased phase−amplitude coupling modulation indices in parieto-occipital brain regions compared to frontal brain regions are also consistent with results from information-theoretic approaches that have demonstrated impaired feedback communication during anesthetic drug-induced altered arousal states^48^.

Loss of responsiveness occurred at anesthetic concentrations that were not consistent with general anesthesia. This finding suggests that using the loss of behavioral responsiveness to nonsalient stimuli as a useful shorthand for unconsciousness should be avoided as it may continue to engender considerable confusion. We note that lack of responsiveness based on epidemiological-based characterizations of general anesthesia does not explicitly preclude the absence of mental content (i.e., mental content may be present but not recalled due to amnestic drug properties). Further, we demonstrate that recovery from anesthesia does not neurophysiologically approximate the baseline state. The neurophysiological differences between emergence and baseline states may explain the anterograde amnesia associated with emergence from sevoflurane anesthesia and the dissociation associated with emergence from sevoflurane plus ketamine anesthesia. Future studies may benefit from airway instrumentation to enable graded increases in the sevoflurane concentration and mechanically controlled ventilation, and methodological advances, such as phase−amplitude coupling estimation methods that model the entire spectrum based on a probabilistic framework to improve the signal to noise ratio of phase−amplitude coupling estimates. These methods may dynamically adjust the driver frequency bandwidth based on data-driven models, improve the temporal resolution associated with calculating phase−amplitude coupling estimates, and offer new insights based on directionality estimation^49^.

**Fig. 5:**
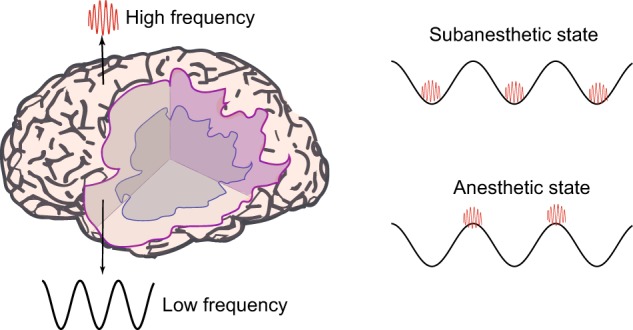
Schematic depicting different cortical origins of low and high-frequency oscillations and the dominant regions of phase-amplitude coupling. During the sevoflurane subanesthetic state, higher frequency activity is limited to the trough of delta oscillations. On the contrary, during sevoflurane general anesthetic states, higher frequency activity is limited to the peak of delta oscillations. Although this dynamic was generated by an anesthetic drug, the present finding suggests that anesthetic states may be produced by extracranial perturbations such as direct current stimulations that cause delta-higher frequency phase−amplitude interactions. Subcortical sources of low-frequency oscillations such as the thalamus is not shown

We reveal a phase−amplitude coupling mechanism between delta and higher frequency oscillations and suggest that this mechanism is functionally and behaviorally relevant for sevoflurane-induced anesthetic states (Fig. 5). We further show that this phase−amplitude coupling mechanism may be conserved across various anesthetic drug classes. Our results are clinically relevant in three key aspects. First, they provide insights into underappreciated mechanisms of anesthetic action and corroborate information processing theories of phase−amplitude coupling. Second, they help define neural oscillatory dynamics for anesthetic brain state monitoring in the operating room and intensive care units, and perhaps, for prognosticating recovery from pathological states of altered arousal. Third, they suggest that anesthetic states may be produced by extracranial stimulations such as transcranial alternating currents that cause delta-higher frequency phase−amplitude interactions. The feasibility of this approach is supported by a recent report that used extracranial stimulations to produce theta-gamma phase−amplitude coupling and improved working memory in humans^50^. This potentially nondrug-mediated approach may fundamentally advance anesthetic care by eliminating cardiovascular and respiratory morbidity that result from off-target drug binding.

## Methods

The Partners Institutional Review Board approved this human research study. This study was registered on www.ClinicalTrials.gov (Identifier: NCT03503578).

### Subject recruitment

This was a single-site, randomized, cross-over study conducted in healthy subjects. Subjects underwent a complete medical history and a pre-anesthesia assessment. The primary inclusion criterion was meeting American Society of Anesthesiology Physical Status I. Key criteria for exclusion were pregnancy, personal or family history of anesthesia-related complications, suspected history of drug abuse, and neuropsychiatric diagnoses. We performed the following screening laboratory tests: complete blood count, liver function, basic metabolic panel, urine toxicology, and urine pregnancy for females. We also recorded data from a 12-lead electrocardiogram. We obtained written informed consent from 12 subjects (7 males), mean age 25 (SD ± 4.7) years, mean weight 70 (11) kg, and mean BMI 24.1 (3) kg/m^2^. We also reanalyzed data from a previously reported study of dexmedetomidine anesthesia^11^. Briefly, dexmedetomidine was administered intravenously as a bolus of 1 μg kg^−1^ over 10 min followed by a 0.7 µg^−1^ kg^−1^ h infusion for 50 min to healthy volunteers, 18−36 years of age. The EEG data analyzed were obtained during the 0.7 µg^−1^ kg^−1^ h maintenance phase of dexmedetomidine.

### Data acquisition

All study procedures were conducted at MGH, Boston, MA. We induced and allowed recovery from sevoflurane general anesthesia and sevoflurane-plus-ketamine general anesthesia in 12 subjects using a cross-over study design (Fig. 1a). Thus, each subject received both sevoflurane-induced general anesthesia (Fig. 1b, c) and sevoflurane-plus-ketamine-induced general anesthesia (Fig. 1d, e) on study visits separated by at least 48 h. Subjects were required to avoid food and water intake for at least 8 h prior to study onset. We reperformed urine toxicology screening, and urine pregnancy testing prior to the administration of sevoflurane. We monitored blood pressure using a standard noninvasive cuff, oxygen saturation using pulse oximetry, ventilation using capnography, and circulation using a 5-lead electrocardiogram.

We instructed all subjects to close their eyes throughout the data acquisition period. Eye closure reduces blinks, muscle artifacts and helps to distinguish between awake occipital alpha oscillations and sevoflurane-induced alpha oscillations. A tight facemask was applied, and subjects were acclimatized to spontaneously breathing through the tight face mask before initiation of the study. We administered sevoflurane using the Dräger Fabius Tiro (Telford, PA, USA) machine. The sevoflurane concentration was analyzed using a General Electric standard multigas module analyzer in clinical use at our institution. During the sevoflurane-induced general anesthesia visit, after 10 min of baseline (awake) recordings, we increased the end-tidal sevoflurane concentration in a stepwise fashion to subanesthetic (1.1%), general anesthetic (2.1%), and deep-general anesthetic (2.8%) states. Each concentration level was maintained for 15 min. During the sevoflurane-plus-ketamine-induced general anesthesia visit, after 10 min of baseline (awake) recordings, we increased the sevoflurane end-tidal concentration to a general anesthetic (2.1%) state and maintained the anesthetic concentration for 45 min. We administered an intravenous bolus of ketamine (0.75 mg^−1^ kg) after achieving 15 min steady-state sevoflurane concentration. Additionally, we recorded 10 min of emergence (awake) EEG data during both study visits. Two board-certified anesthesiologists were present during all study procedures.

We recorded high-density EEG signals using the Waveguard system with a standard EEG cap (64 channels, ANT Neuro, Netherlands) and electrode impedances of <5 kΩ. We instructed subjects to click a mouse button when they heard auditory stimuli. The auditory stimuli were either a verbal command to press a button or auditory steady-state responses (40 and 80 Hz). We randomly presented auditory stimuli every 4–8 s. All auditory stimuli were 1 s long and were delivered using headphones (ER2; Etymotic Research).

### Data preprocessing and epoch selection

We down-sampled the EEG data to 250 Hz, spline-interpolated corrupted data, and remontaged the data using a nearest-neighbor Laplacian referencing scheme. For the sevoflurane visit, we selected 5-min EEG epochs. These EEG epochs were selected 10 min after the sevoflurane concentration reached the desired steady-state concentrations of 1.1, 2.1, and 2.8%. For the sevoflurane-plus-ketamine visit, we selected 5-min EEG epochs. These EEG epochs were selected 10 min after sevoflurane reached the desired steady-state concentration of 2.1% and 2 min after the ketamine bolus was administered. We also selected 5-min EEG epochs during baseline and emergence periods.

For spectral analyses, we averaged data from three channels to approximate frontal channel location corresponding to Fz. We also averaged data from three channels to approximate occipital channel location corresponding to Pz. Two investigators visually inspected the selected EEG data to ensure artifact-free epochs.

### Probability of response analysis

Button press responses to auditory stimuli were binarized. We computed subject level probability (response) curves from the binary response data with a Bayesian state-space algorithm^51^. We defined the loss of responsiveness (LOR) as the probability (response) <0.05 after the administration of sevoflurane and if maintained for at least 5 min. We defined the return of responsiveness (ROR) as the probability (response) >0.05 after the discontinuation of sevoflurane and if maintained for at least 5 min.

### Spectral analysis and spatial plots

We used the Chronux toolbox in Matlab 2018 (Mathworks, Natick, MA) to compute multitaper spectral estimates. The parameters used for statistical inference were: TW or time-bandwidth product = 3, *K* or number of tapers = 5, *T* or window size = 2 s, and no overlapping windows. We computed the median of the spectral estimate across all subjects for group-level visualization. We also computed the median of the group averaged spectral estimates over time and frequency bands of interest. The topoplot function in EEGLab was used to interpolate these data and to generate spatial head plots.

### Global coherence analysis and spatial plots

Global coherence is a multivariate measure of synchrony. We estimated global coherence for all electrode locations except the bilateral mastoids and electrooculogram. First, we computed the cross-spectral matrix for every subject over every nonoverlapping moving window using multitaper methods. Next, we computed the median of the imaginary and real components of the cross-spectral matrix. Finally, for each frequency and time window, we computed the singular value decomposition of the cross-spectral matrix. We normalized the dominant singular value by the trace of the singular value matrix to obtain global coherence for a given frequency and time window. We computed the median global coherence estimates across all subjects for group-level visualization. We also computed the median singular vector across subjects for a given frequency of interest. We scaled this vector with the median of the group averaged singular value over time for the given frequency of interest. The topoplot function in EEGLab was used to interpolate the data and to generate spatial head plots.

### Modulation index

The modulation index, an adaptation of the Kullback–Leibler distance, is a scalar measure of phase−amplitude coupling between two frequency ranges of interest: phase modulating (phase driver) and amplitude modulated frequency bands^41^. However, the modulation index does not make clear the phase−amplitude coupling relationship between the phase modulating and the amplitude modulated frequency bands (i.e., whether the amplitude-modulated band resides on the peak or trough of the phase-modulated band cannot be deciphered from the modulation index). The phaseampogram is used to reveal the phase−amplitude relations between the phase-modulating and amplitude-modulated frequency bands^6^. The comodulogram, an extension of the modulation index, is a principled approach to choosing the phase-modulating frequency band and amplitude-modulated frequency bands of interest. This is because the comodulogram depicts modulation indices across a range of phase-modulating and amplitude-modulated frequency bands^41^. We computed the comodulogram on 60-s long nonoverlapping data segments. These data were averaged to obtain a within-subject comodulogram. We next computed the median comodulogram across subjects. We calculated the modulation index between lower (0.1–8 Hz, 0.32 Hz steps, 1 Hz bandwidth) and higher frequencies (1–30 Hz, 1 Hz steps, 2 Hz bandwidth) as previously described^41^.

### Phase−amplitude coupling and amplitude vector distribution

Phase−amplitude coupling and amplitude vector distribution were computed on 60-s long, nonoverlapping data segments. These data were averaged to obtain within-subject estimates. We next computed the median across subjects.

To compute phase−amplitude coupling dynamics associated with low and high frequencies of interest, we constructed phaseampograms by adapting a previously described method to our dataset^41^. We calculated the phaseampograms between delta frequencies (2–4 Hz) and higher frequencies (4.1–30 Hz, 3 Hz steps). We computed the median phaseampogram across all subjects for group-level visualization.

We computed the normalized mean amplitude vector corresponding to the higher frequencies of interest and the circular mean of the mean amplitude vectors. This resulted in a vector whose length represents the mean amplitude of higher frequency activity and phase represents the mean angular location of higher frequency on the phase of the low oscillation driver frequency. We computed the mean amplitude vector for group-level visualization.

### Statistics and reproducibility

We used an empirical bootstrap approach to enable statistical inferences^52^. First, we bootstrapped the estimates of each nonoverlapping window. Next, we computed the median of the bootstrapped estimates at the subject level and computed the group median of this estimate. We computed the median difference between groups and then iterated the above procedure 5000 times to obtain a distribution of the median difference between groups. We computed the 99% confidence interval of this distribution. We used the omnibus circular test statistic to test phase uniformity^53^. We performed a linear mixed-effects model with Tukey’s HSD for post-hoc comparisons to test for differences between the mean amplitude vectors JMP®, Pro 14 (SAS Institute Inc., Cary, NC, 1989–2007).

### Reporting summary

Further information on research design is available in the Nature Research Reporting Summary linked to this article.

## Supplementary information

Supplementary Information

Supplementary Data 1

Description of Additional Supplementary Files

Supplementary Movie 1

Supplementary Movie 2

Reporting Summary

## Data Availability

The data supporting the findings of this study will be made available from the corresponding authors upon reasonable request.
