# Supplementary material for: Delta oscillations phase limit neural activity during sevoflurane anesthesia
Source: Commun Biol. 2019 Nov 15;2:415. doi: 10.1038/s42003-019-0664-3 (PMC6858348; doi:10.1038/s42003-019-0664-3)
Supplement: Supplementary file 1 — Supplementary Information [file 42003_2019_664_MOESM1_ESM.pdf]

Supp. Fig. 1

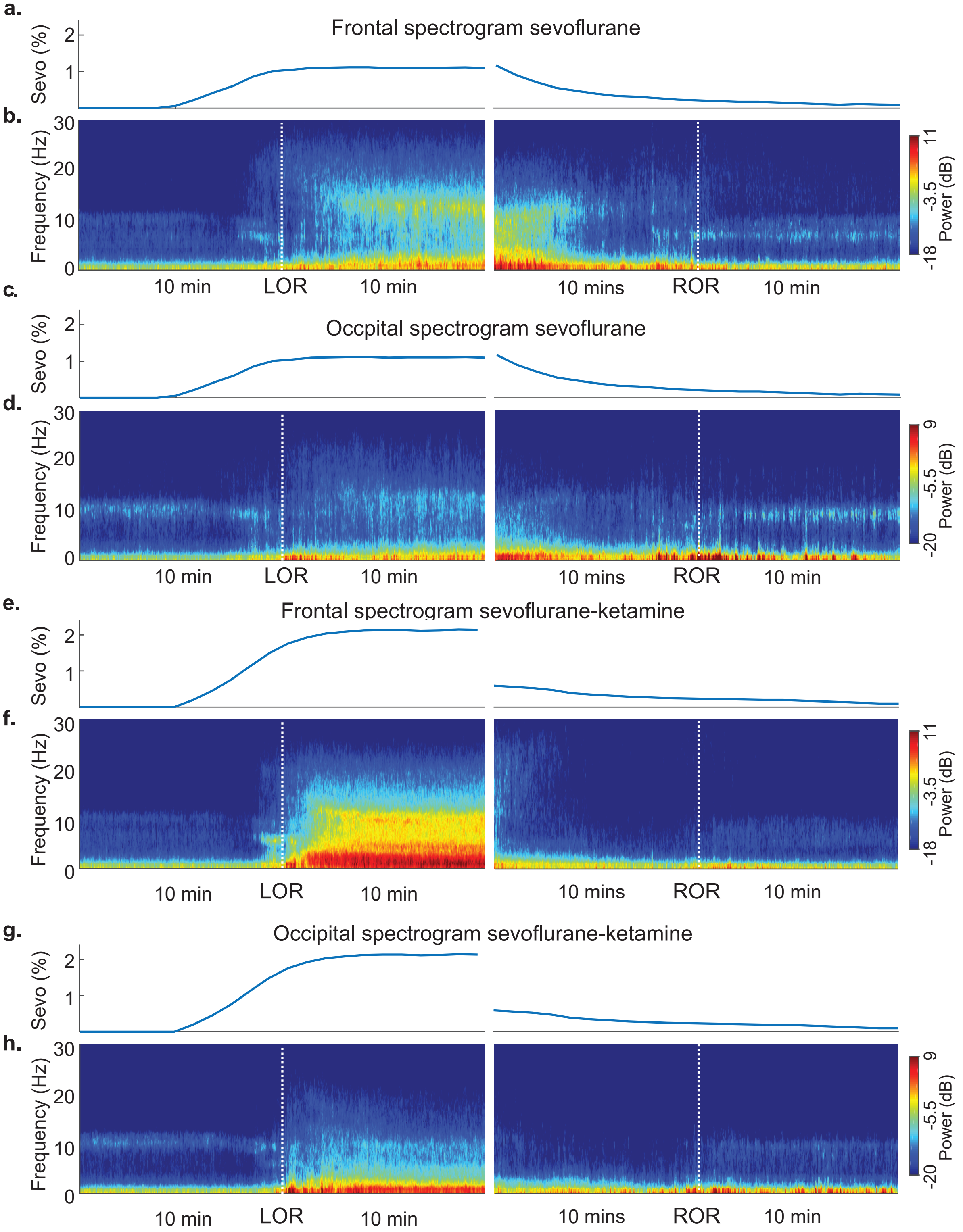

Electroencephalogram data aligned to (±10 minutes) loss and recovery of responsiveness. **a, b** Median group-level sevoflurane concentration and corresponding median frontal spectrogram during the sevoflurane visit. Median group-level end tidal sevoflurane concentration observed at LOR was higher than median group-level end tidal sevoflurane concentration observed at ROR. **c, d** Median group-level sevoflurane concentration and corresponding median occipital spectrogram. **e, f** Median group-level sevoflurane concentration and corresponding median frontal spectrogram during the sevoflurane-plus-ketamine visit. **g, h** Median group-level sevoflurane concentration and corresponding median occipital spectrogram during the sevoflurane-plus-ketamine visit.

LOR, loss of responsiveness. ROR, return of responsiveness. White dotted lines represent behavioral state transitions.

Supp. Fig. 2

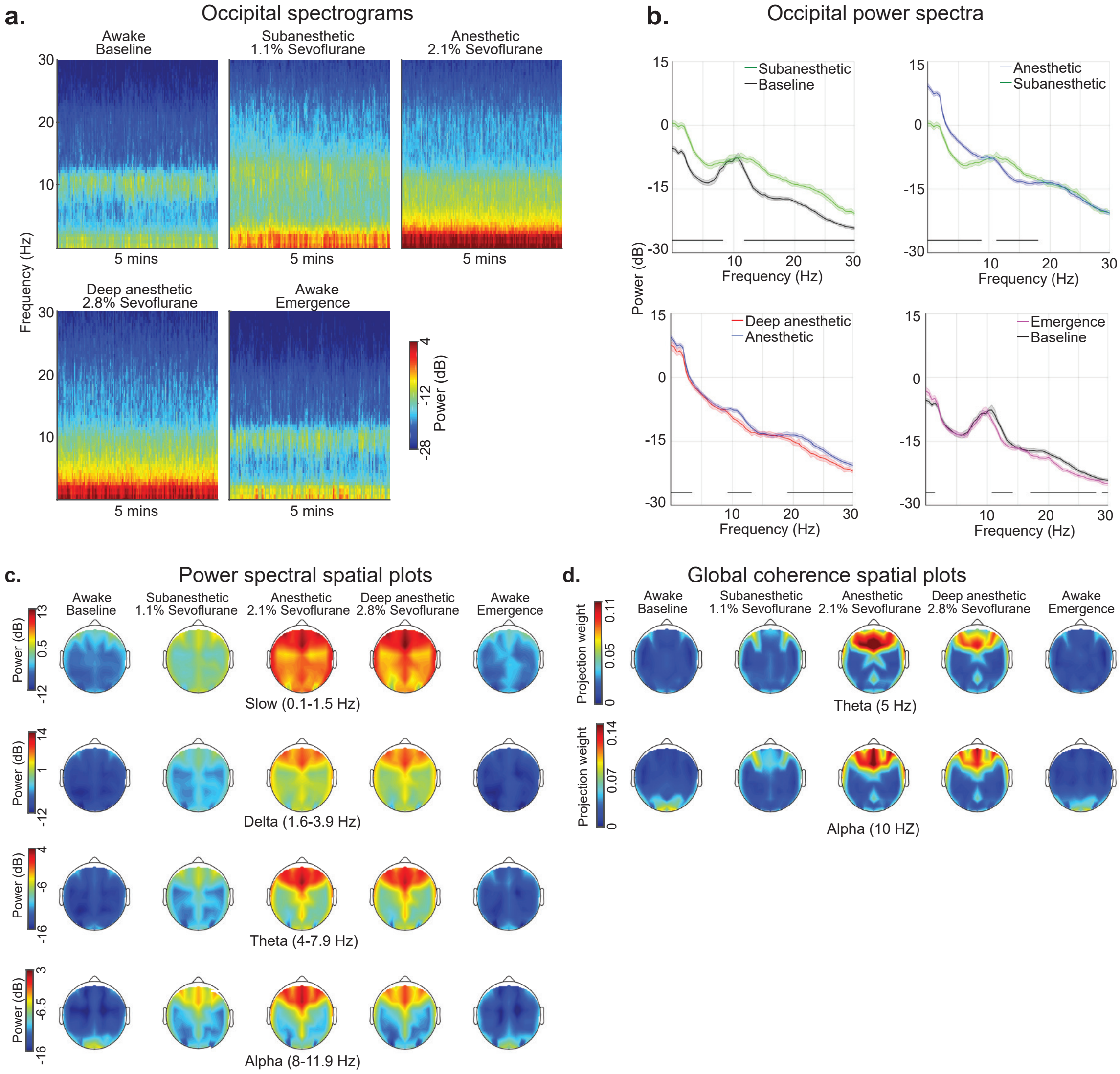

Occipital spectrograms, spectra, and spatial plots during the sevoflurane visit. **a** The median occipital spectrograms demonstrated that electroencephalogram oscillations change systematically as a function of the anesthetic state. **b** Occipital spectra and bootstrapped difference of median spectra confirm that electroencephalogram oscillations change systematically as a function of the anesthetic state. Different from the frontal analysis, an increase in slow oscillation power did not covary with anesthetic depth. Similar to the frontal analysis, alpha oscillation power did not covary with the anesthetic depth. **c** Power spectral-spatial plots demonstrated that slow oscillations were increased in power and were spatially distributed across electrode locations during the anesthetic states. Delta, theta, and alpha oscillations were also increased in power but with a frontal dominance. **d** Global coherence spatial plots demonstrated that theta and alpha oscillations were globally coherent with frontal dominance.

Shaded regions represent the 99% confidence bounds of the bootstrapped median power spectra. Black lines represent frequency bands that met our threshold for statistical significance.

Supp. Fig. 3

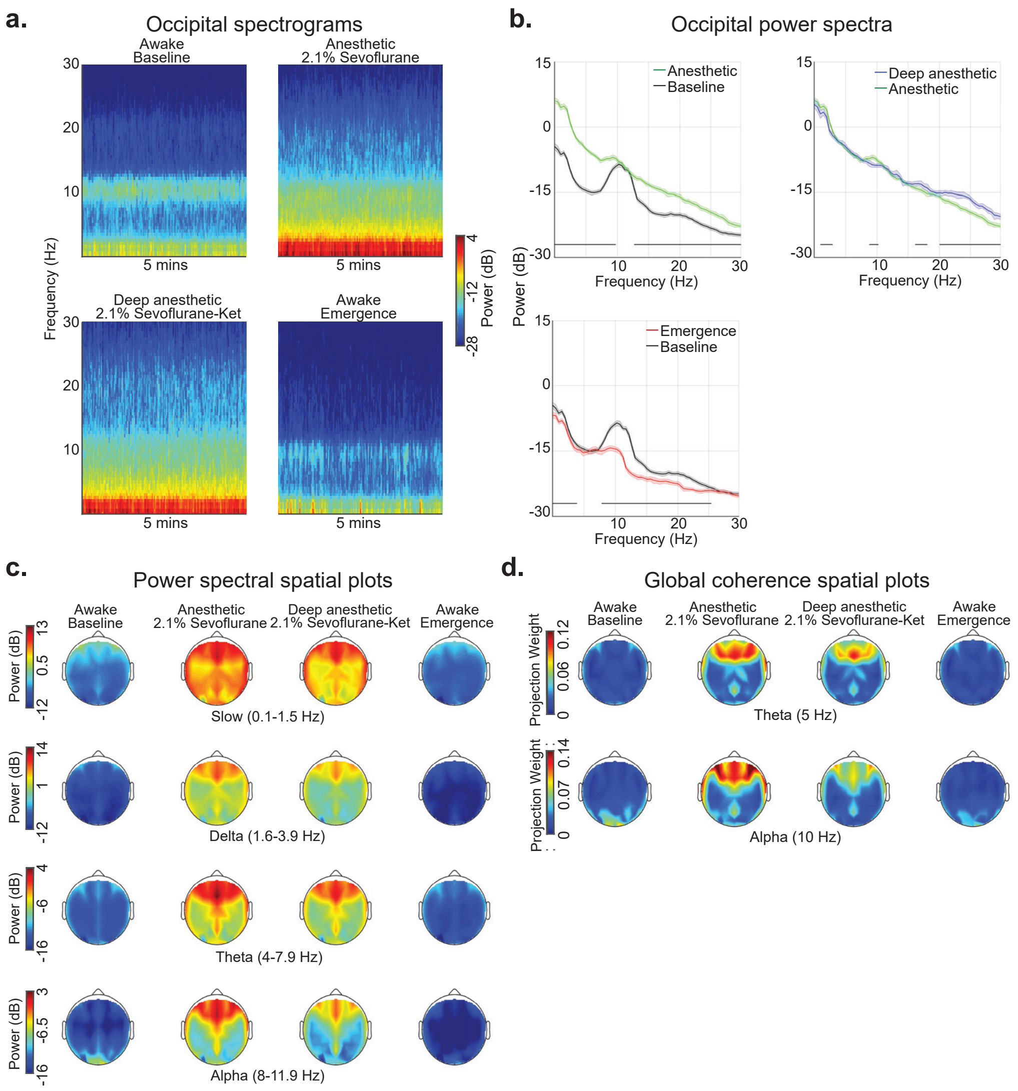

Occipital spectrograms, spectra, and spatial plots during the sevoflurane-plus-ketamine visit. **a** The median occipital spectrograms demonstrated that electroencephalogram oscillations change systematically as a function of the anesthetic state. **b** Occipital spectra and bootstrapped difference of median spectra confirm that electroencephalogram oscillations change systematically as a function of the anesthetic state. Alpha oscillation power did not covary with the anesthetic depth. **c** Power spectral-spatial plots demonstrated that ketamine reduced the power of frontally dominant alpha oscillations. **d** Global coherence spatial plots demonstrated that ketamine reduced the coherence of theta and alpha oscillations.

Shaded regions represent the 99% confidence bounds of the bootstrapped median power spectra. Black lines represent frequency bands that met our threshold for statistical significance.

Supp. Fig. 4

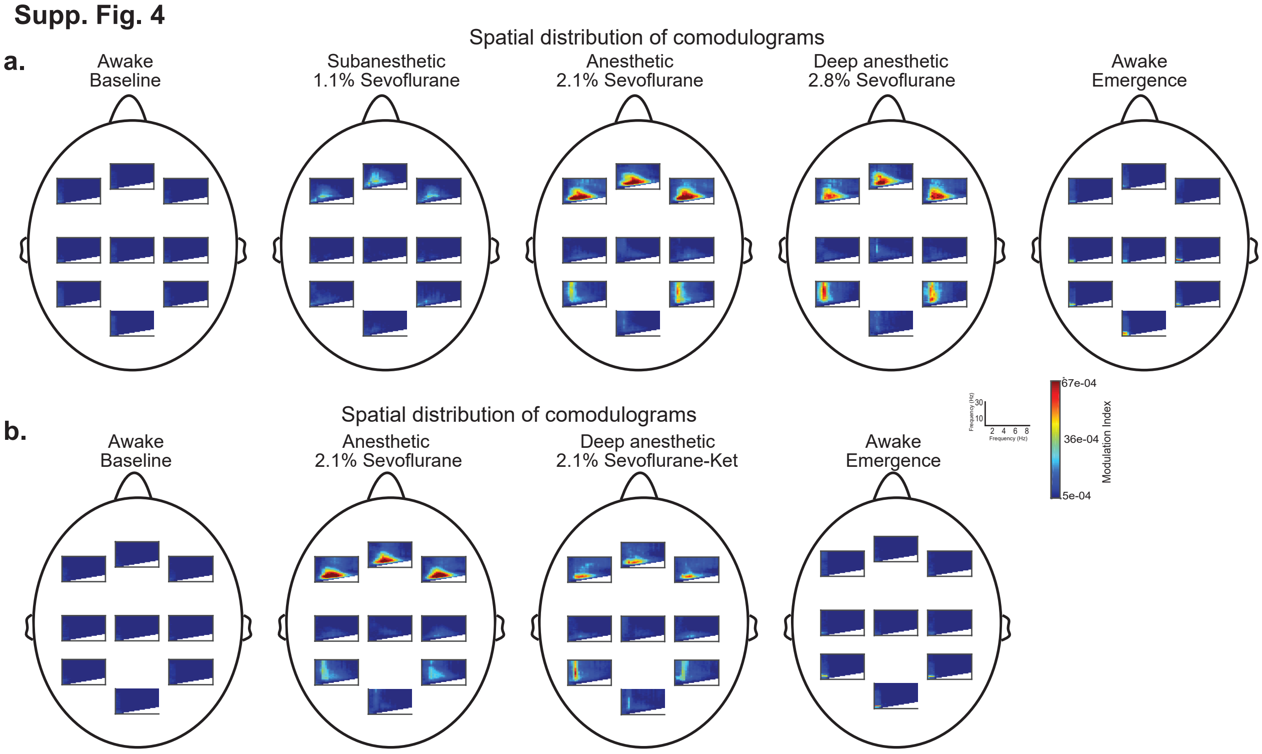

Spatial distribution of comodulograms. **a, b** Comodulograms demonstrated that frontal regions had higher delta oscillation modulation indices compared to parieto-occipital regions. Modulation indices were increased from baseline during the anesthetic states.

Supp. Fig. 5

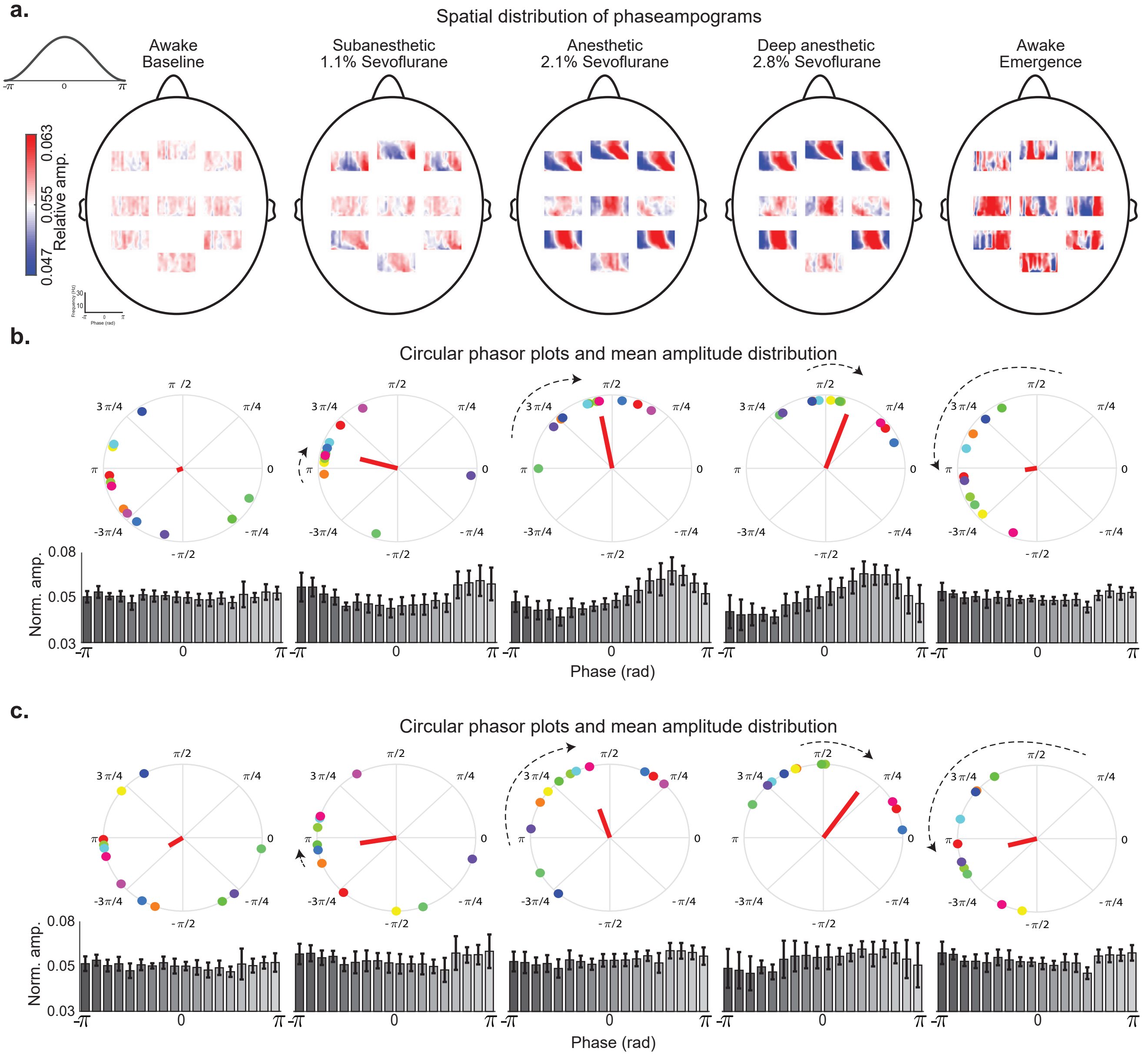

Spatial distribution of phase-amplitude coupling dynamics during the sevoflurane visit. **a** Phaseampograms between delta and higher frequencies demonstrated that distinct patterns of phase limited neural activity associated with subanesthetic and anesthetic states were conserved across brain regions. Frontal regions demonstrated higher relative amplitude at approximately 0 phase region of delta oscillations. During the subanesthetic state, the occipital region was associated with higher relative amplitude at approximately 0 phase, while the frontal region was associated with higher relative amplitude at approximately π phase. **b** Frontal circular phasor plots of delta (1.6-3.9 Hz) demonstrated that alpha (8-11.9 Hz) oscillation activity systematically shifted from π towards 0 phase of delta oscillations as a function of anesthetic depth. The median amplitude vector (red line) was increased from baseline during the anesthetic states. Mean amplitude distribution was not uniformly distributed during sevoflurane-induced anesthetic states. **c** Frontal circular phasor plots of slow (0.1-1.5 Hz) oscillations demonstrated that alpha oscillation activity also systematically shifted from π towards 0 phase of delta oscillations as a function of anesthetic depth. The median amplitude vector (red line) was increased from baseline during the anesthetic states. However, worse signal to noise ratio can be observed in the mean amplitude distribution to suggest that an appropriately chosen phase driver frequency band is important.

Colored circular dots on phasor plots represent subject level data. Error bars represent standard deviation.

Supp. Fig. 6

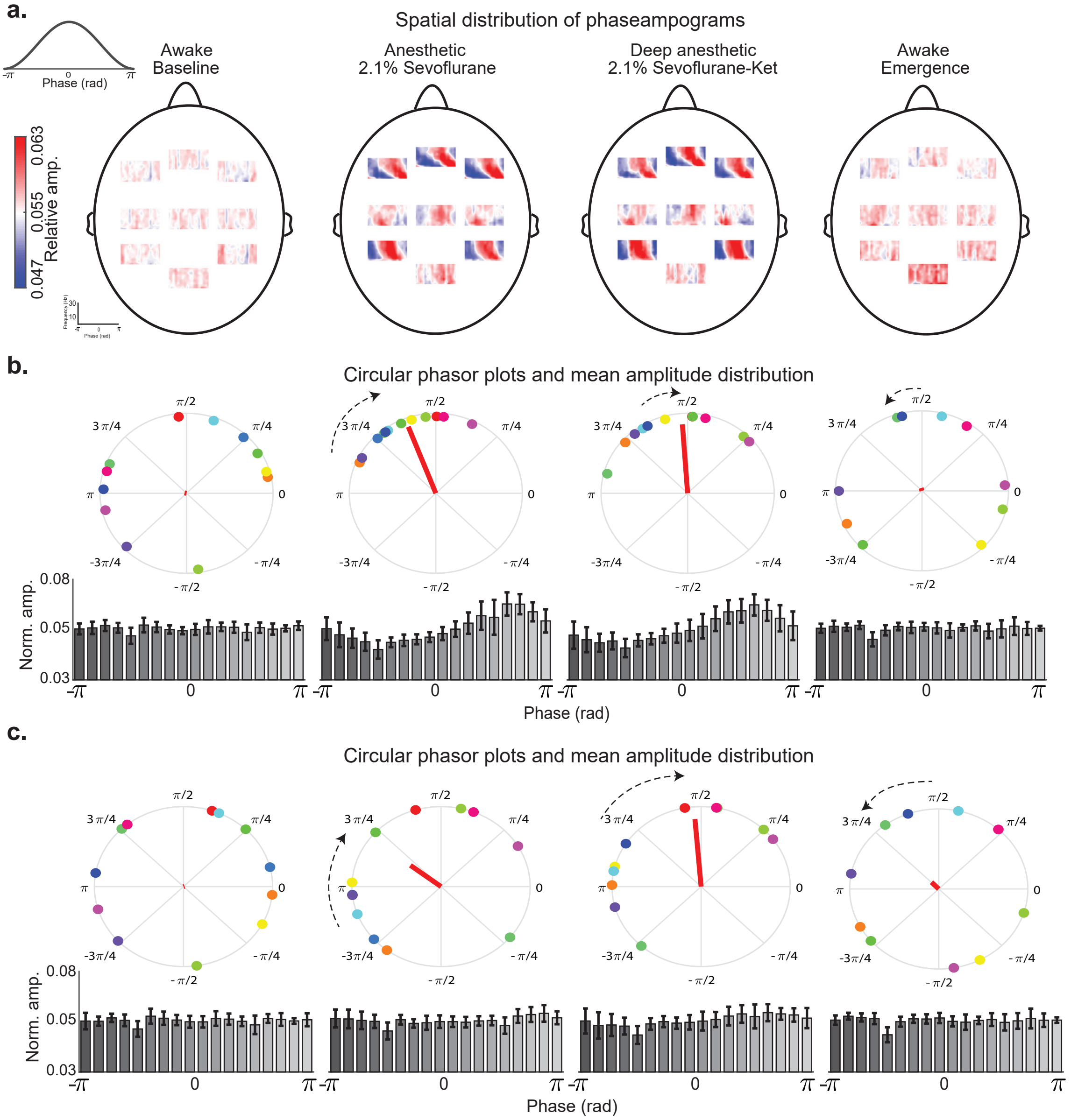

Spatial distribution of phase-amplitude coupling dynamics during the sevoflurane-plus-ketamine visit. **a** Phaseampograms between delta and higher frequencies demonstrated that distinct patterns of phase limited neural activity associated with subanesthetic and anesthetic states were conserved across brain regions. **b** Frontal circular phasor plots of delta (1.6-3.9 Hz) demonstrated that alpha (8-11.9 Hz) oscillation activity systematically shifted from π towards 0 phase of delta oscillations as a function of anesthetic depth. The median amplitude vector (red line) was increased from baseline during the anesthetic states. Mean amplitude distribution was not uniformly distributed during sevoflurane-induced anesthetic states. **c** Frontal circular phasor plots of slow (0.1-1.5 Hz) oscillations demonstrated that alpha oscillation activity also systematically shifted from π towards 0 phase of delta oscillations as a function of anesthetic depth. The median amplitude vector (red line) was increased from baseline during the anesthetic states. However, worse signal to noise ratio can be observed in the mean amplitude distribution to suggest that an appropriately chosen phase driver frequency band is important.

Colored circular dots on phasor plots represent subject level data. Error bars represent standard deviation.

Supp. Fig. 7

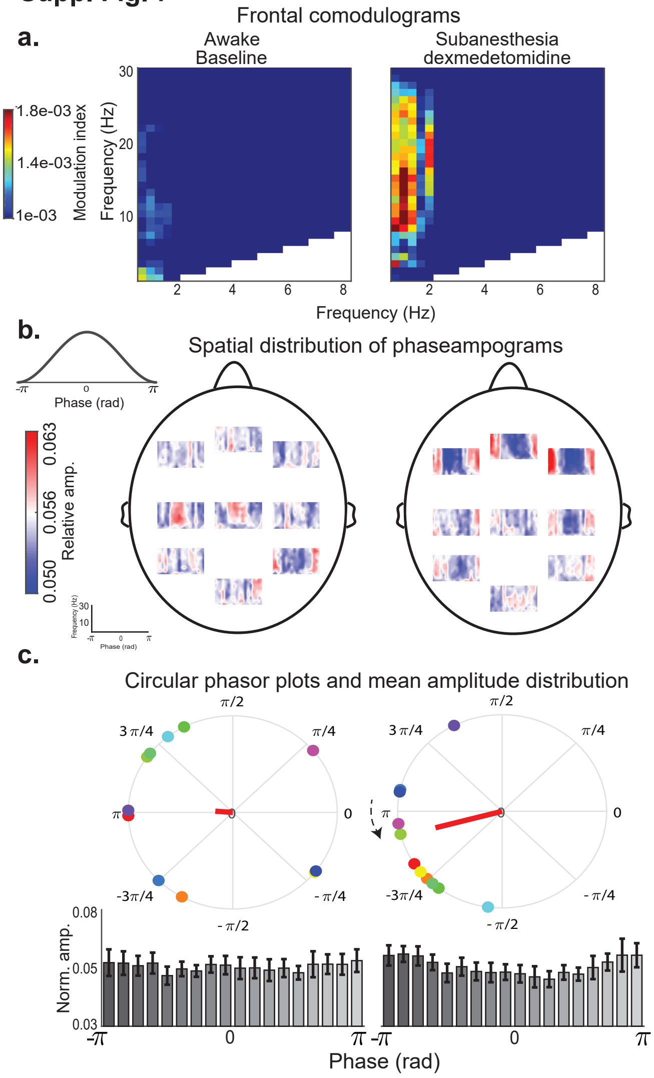

Phase-amplitude coupling dynamics associated with dexmedetomidine. **a** Frontal comodulograms demonstrated that slow (0.1-1.5 Hz) oscillations modulated higher frequencies during the dexmedetomidine subanesthetic state. **b** Phaseampograms between slow and higher frequencies demonstrated that distinct patterns of phase limited neural activity associated with the dexmedetomidine subanesthetic state. Similar to the sevoflurane subanesthetic state, the occipital region was associated with higher relative amplitude at approximately 0 phase, while the frontal region was associated with higher relative amplitude at approximately π phase. **c** Frontal circular phasor plots of slow oscillations demonstrated that although spindle (13-16 Hz) oscillation activity remained approximately at π phase during the dexmedetomidine subanesthetic state, the median amplitude vector (red line) was increased from baseline. Mean amplitude distribution was not uniformly distributed during the sevoflurane-induced subanesthetic state. Colored circular dots on phasor plots represent subject level data. Error bars represent standard deviation.
