# Supplementary material for: Delta oscillations phase limit neural activity during sevoflurane anesthesia
Source: Commun Biol. 2019 Nov 15;2:415. doi: 10.1038/s42003-019-0664-3 (PMC6858348; doi:10.1038/s42003-019-0664-3)
Supplement: Supplementary file 3 — Description of Additional Supplementary Files [file 42003_2019_664_MOESM3_ESM.docx]

Description of Additional Supplementary Items

Supplementary Data 1: Source Data for figure 2b, 2e, 3b and 3e

Supplementary Movie 1: Illustrates the oscillatory and global coherence changes associated with sevoflurane anesthetic states.

Supplementary Movie 2: Illustrates the oscillatory and global coherence changes associated with sevoflurane anesthetic states.
