# Supplementary material for: Delta oscillations phase limit neural activity during sevoflurane anesthesia
Source: Commun Biol. 2019 Nov 15;2:415. doi: 10.1038/s42003-019-0664-3 (PMC6858348; doi:10.1038/s42003-019-0664-3)
Supplement: Supplementary file 6 — Reporting Summary [file 42003_2019_664_MOESM6_ESM.pdf]

nature research

Corresponding author(s): Shubham Chamadia
Last updated by author(s): Oct 18, 2019

Reporting Summary

Nature Research wishes to improve the reproducibility of the work that we publish. This form provides structure for consistency and transparency in reporting. For further information on Nature Research policies, see Authors & Referees and the Editorial Policy Checklist.

Statistics

For all statistical analyses, confirm that the following items are present in the figure legend, table legend, main text, or Methods section.

n/a	Confirmed	
☐	☒	The exact sample size (n) for each experimental group/condition, given as a discrete number and unit of measurement
☐	☒	A statement on whether measurements were taken from distinct samples or whether the same sample was measured repeatedly
☐	☒	The statistical test(s) used AND whether they are one- or two-sided<br>*Only common tests should be described solely by name; describe more complex techniques in the Methods section.*
☒	☐	A description of all covariates tested
☒	☐	A description of any assumptions or corrections, such as tests of normality and adjustment for multiple comparisons
☐	☒	A full description of the statistical parameters including central tendency (e.g. means) or other basic estimates (e.g. regression coefficient) AND variation (e.g. standard deviation) or associated estimates of uncertainty (e.g. confidence intervals)
☒	☐	For null hypothesis testing, the test statistic (e.g. F, t, r) with confidence intervals, effect sizes, degrees of freedom and P value noted<br>*Give P values as exact values whenever suitable.*
☒	☐	For Bayesian analysis, information on the choice of priors and Markov chain Monte Carlo settings
☒	☐	For hierarchical and complex designs, identification of the appropriate level for tests and full reporting of outcomes
☒	☐	Estimates of effect sizes (e.g. Cohen's d, Pearson's r), indicating how they were calculated

Our web collection on statistics for biologists contains articles on many of the points above.

Software and code

Policy information about availability of computer code

Data collection	Dräger Fabius Tiro (Telford, PA, USA) machine, Waveguard system 64 channels EEG cap (ANT Neuro, Netherlands).
Data analysis	Data analyses were performed using the open source Chronux toolbox in Matlab 2018 (Mathworks, Natick, MA).

For manuscripts utilizing custom algorithms or software that are central to the research but not yet described in published literature, software must be made available to editors/reviewers. We strongly encourage code deposition in a community repository (e.g. GitHub). See the Nature Research guidelines for submitting code & software for further information.

Data

Policy information about availability of data

All manuscripts must include a data availability statement. This statement should provide the following information, where applicable:

- Accession codes, unique identifiers, or web links for publicly available datasets
- A list of figures that have associated raw data
- A description of any restrictions on data availability

The authors declare that the majority of data supporting the findings of this study are available within the paper and its supplementary information files. All relevant data supporting the findings of this study are available from the corresponding author upon reasonable request.

Field-specific reporting

Please select the one below that is the best fit for your research. If you are not sure, read the appropriate sections before making your selection.

☒ Life sciences ☐ Behavioural & social sciences ☐ Ecological, evolutionary & environmental sciences

Life sciences study design

All studies must disclose on these points even when the disclosure is negative.

Sample size	We collected and analyzed the EEG data from 12 healthy subjects undergoing anaesthetic administration.
Data exclusions	Due to hardware malfunctioning, we discarded the missing data.
Replication	To confirm result replicability, we implemented bootstrap approach where a subset of data points were analyzed and iterated over n=5000. iterations.
Randomization	Our study involves randomized, cross-over study design that is also illustrated as a figure 1.
Blinding	The investigators were not blinded to the group allocation as the analysis was initiated after the complete collection of data.

Reporting for specific materials, systems and methods

We require information from authors about some types of materials, experimental systems and methods used in many studies. Here, indicate whether each material, system or method listed is relevant to your study. If you are not sure if a list item applies to your research, read the appropriate section before selecting a response.

Materials & experimental systems

n/a	Involved in the study	
☒	☐	Antibodies
☒	☐	Eukaryotic cell lines
☒	☐	Palaeontology
☒	☐	Animals and other organisms
☐	☒	Human research participants
☐	☒	Clinical data

Methods

n/a	Involved in the study	
☒	☐	ChIP-seq
☒	☐	Flow cytometry
☒	☐	MRI-based neuroimaging

Human research participants

Policy information about studies involving human research participants

Population characteristics	We obtained written informed consent from 12 healthy subjects (7 males), mean age 25 (SD ± 4.7) years, mean weight 70 (11) kg, and mean BMI 24.1 (3) kg/m2.
Recruitment	The criteria used for selection was that the subjects needed to be at between 18-50 years of age. The primary inclusion criterion was meeting American Society of Anesthesiology Physical Status I. Key criteria for exclusion were pregnancy, personal or family history of anesthesia-related complications, suspected history of drug abuse, and neuropsychiatric diagnoses. We performed the following screening laboratory tests: complete blood count, liver function, basic metabolic panel, urine toxicology, and urine pregnancy for females.
Ethics oversight	This study was approved by the Human Research CommitteeEthics Review Committee of the Massachusetts General Hospital (IRB Identifier: NCT03503578) and an informed consent was obtained from the patient

Note that full information on the approval of the study protocol must also be provided in the manuscript.

Clinical data

Policy information about clinical studies

All manuscripts should comply with the ICMJE guidelines for publication of clinical research and a completed CONSORT checklist must be included with all submissions.

Clinical trial registration	Identifier: NCT03503578
Study protocol	https://clinicaltrials.gov/ct2/show/NCT03503578?cond=NCT03503578&rank=1
Data collection	https://clinicaltrials.gov/ct2/show/NCT03503578?cond=NCT03503578&rank=1
Outcomes	Change in multitaper spectral estimates of EEG power during sevoflurane-induced general anesthesia
